# The Salience of Trust to the Client-Provider Relationship in Post-Ebola Guinea: Findings From a Qualitative Study

**DOI:** 10.9745/GHSP-D-21-00429

**Published:** 2022-02-28

**Authors:** Natalie Tibbels, Zoé Hendrickson, Hannah Mills, Sidikiba Sidibé, Claudia Vondrasek, Tilly Gurman

**Affiliations:** a Johns Hopkins Bloomberg School of Public Health, Center for Communication Programs, Baltimore, MD, USA.; b Johns Hopkins Center for Communication Programs-Guinea, Conakry, Guinea.

## Abstract

Lack of trust in the health care system can serve as a barrier to service utilization, especially in pandemic and postemergency settings. Although previous research has identified domains of trust that contribute to individuals’ trust in the health system, little research exists from low- and middle-income countries, particularly during and after infectious disease outbreaks. The current study—conducted to inform activities for a post-Ebola program—explored perceptions and experiences of health care provision in post-Ebola Guinea, with particular attention to trust. Researchers conducted in-depth interviews with health workers (n=15) and mothers of young children (n=29) along with 12 focus group discussions with grandmothers of young children and 12 with male heads of household. The study occurred in Basse Guinée and Guinée Forestière—2 areas hardest hit by Ebola. Respondents identified a breach of trust during the epidemic, with several domains emerging as relevant for renewed trust and care-seeking practices. At the core of a trusting client-provider relationship was the inherent belief that providers had an intrinsic duty to treat clients well. From there, perceived provider competence, the hospitality at the facility, provider empathy, transparency about costs, and commitment to confidentiality emerged as relevant influences on participant trust in providers. Community members and providers expressed similar viewpoints regarding trust and discussed the role of open communication and community mobilization in rebuilding trust. Study findings informed a variety of program activities, including the development of campaign messages and interpersonal communication trainings for health workers. This study provides valuable insight about some underlying components of trust that can provide key leverage points to rebuild trust and promote care seeking in postemergency settings. This insight is informing program activities in the current Ebola response in Guinea and could be useful in other crises, such as the global coronavirus disease (COVID-19) pandemic.

## Abstract (precis)

This qualitative study in post-Ebola Guinea showed that trust was a salient construct for clients making health care-seeking decisions in a postemergency setting. This analysis argues for global health programs to build trust between clients and the health system by addressing underlying domains of trust as defined by the clients themselves.

## Teaser

**Key Findings:**

- Trust is a vital determinant of health care seeking and should be a key consideration for global health programs operating in postemergency settings.
- Trust is a composite of various underlying domains, and rebuilding trust depends on understanding and addressing the domains that are salient to clients.
- This analysis provides a case study of how a global health program used qualitative research to explore salient domains of trust within the client-provider relationship in post-Ebola Guinea and developed interventions to address those domains.

**Key Implications:**

- To improve care-seeking practices where trust barriers exist between communities and the health system, program managers should seek to center the clients by defining and addressing their conception of trust.

Résumé en français à la fin de l’article.

## INTRODUCTION

As the coronavirus disease (COVID-19) pandemic rages throughout the world, public health experts have identified the urgent need to build and guard trust between communities and health systems.^1^ Prior infectious disease outbreaks have likewise demonstrated the importance of building trust through risk communication and community engagement. Rapidly applying these lessons is critical for a robust response to COVID-19.^2^

Of particular relevance is the West African Ebola epidemic, which began in rural Guinea in December 2013, and ultimately spread to Conakry by mid-2014, reaching the capitals of Sierra Leone and Liberia by July 2014. Ultimately, there were 28,616 total cases across the 3 countries, with 3,814 cases and 2,544 deaths in Guinea alone.^3^ The recent reemergence of Ebola in Guinea has added complexity to the COVID-19 response, particularly as vaccines for the Ebola virus and the novel severe acute respiratory syndrome coronavirus 2 (SARS-CoV2) are being delivered.^4^

Before 2013, despite policy initiatives to expand and improve access to health care, the Guinean health system faced understaffed and underresourced facilities, significant health disparities, and almost no systematic infectious disease surveillance or control.^5^ Throughout West Africa, international experts and local leaders alike identified a lack of engagement with communities and a lack of trust in the health system as key drivers of the epidemic.^6^^–^^9^ Specifically, mistrust in the health system and health workers can affect individuals’ willingness to seek care or adopt epidemic control measures, leading to delays in care seeking and, consequently, poor health outcomes.^10^^–^^14^ A more in-depth understanding of trust, or lack thereof, should inform efforts to address recurrent Ebola outbreaks in central Africa and strategies for other emergent diseases like COVID-19.

Individual’s mistrust in the health system and health workers can undermine their willingness to seek care or adopt epidemic control measures.

Trust is the voluntary willingness to engage with a person, group, or institution when the outcome is uncertain or there is some inherent condition of risk.^15^^,^^16^ Trust is particularly relevant within the health care context, as there is an inherent power imbalance between providers and clients. Gilson outlined the relationship between trust and power, highlighting that (1) an appropriate measure of distrust on the client side can limit abuses of power; and (2) specific actions or characteristics on the provider side can, by avoiding abuse of power, create the basis for a trusting relationship.^15^ Understanding and overcoming this interpersonal boundary between clients and providers is critical to improving health care outcomes, especially in countries like Guinea.

The Trust Determination Model suggests that trust is determined by perceived competence, perceived honesty, and perceived concern.^17^ Other key domains related to trust include, among others, communication, fairness, and confidentiality.^18^ Trust is a useful concept for exploring the client-provider relationship in post-Ebola Guinea for several reasons. First, trust operates in an environment of uncertainty—an apt description of the Guinean health system during and immediately after the Ebola epidemic despite the heroic efforts of frontline health workers and government officials.^15^ Second, trust is voluntary, suggesting a multiplicity of care options that Guineans access through both the formal and informal health sector.^19^ Third, trust may facilitate health care seeking, which has been a significant programmatic challenge in Ebola recovery efforts^20^ and likely affects care seeking for COVID-19. With all that is known about trust and its role in care seeking, a gap remains; previous research on trust has largely focused on high-income settings, with minimal attention to this topic in West Africa.^15^^–^^18^

With all that is known about trust and its role in care seeking, a gap remains in the literature.

The current study helps to fill this disconcerting gap by exploring constructs related to trust in Guinea. The study sought to identify how men and women in Guinea described their interactions with health workers in health facilities, including the factors that contributed to their trust, or lack of trust, in these settings.

## METHODS

The current study served as formative research for the Health Communication Capacity Collaborative (HC3) project, which worked in post-Ebola Guinea to increase the demand for and use of quality reproductive, maternal, neonatal, and child health (RMNCH) services. Local researchers conducted focus group discussions (FGDs) and in-depth interviews (IDIs) in June 2016 in Basse Guinée and Guinée Forestière, 2 of the areas most affected by Ebola. Data collection occurred in 3 sites per region, including both urban and rural sites (Table 1).

**TABLE 1.** Study Sites, Qualitative Study on RMNCH Service Utilization, Guinea

	**Basse Guinée**	**Guinea Forestère**
Urban	Conakry/Ratoma Télimélé	Nzérékoré
Rural	Friguibegbe	Bendou Kissidougou Senko/Beyla

Abbreviation: RMNCH, reproductive, maternal, neonatal, and child health.

IDIs included individuals directly involved in RMNCH services, either as a client (mothers of children aged younger than 5 years) or as a health care provider (hospital-based doctor, community health center-based nurse-midwife, or community health worker). Mothers needed to be aged 20–40 years, either married or cohabitating with their partner, have at least 1 child aged younger than 5 years. FGDs included individuals that influence service utilization by women, namely grandmothers of children aged younger than 5 years and male heads of household. Grandmothers needed to be aged 45–60 years and the grandmother of at least 1 child aged younger than 5 years. Male heads of household needed to be aged 20–50 years and the head of household with at least 1 child aged younger than 5 years. The sample size was estimated according to time and resource allowances and to be sufficient for triangulation of data per type of qualitative methodology and type of participant.^21^

Two of the lead researchers trained 2 local data collection teams, 1 per site, comprised of investigators with experience in qualitative research. Data collectors worked with community health workers, local leaders, and local organizations to identify eligible households. In each selected household, a member of the study team and 1 of the community-based focal points visited and invited 1 eligible woman to participate in the IDI. Data collectors also worked with the local focal points to recruit eligible grandmothers and male heads of household. Snowball sampling was used in particular cases where participants were willing to refer other community members to participate. Staff at relevant health facilities assisted with recruiting health workers. The research team informed the local health authorities about the study to obtain their support in identifying potential participants. Each recruitment script described the goal of the research study as understanding perceptions and experiences with local health facilities. There were no cases of nonresponse or refusal to participate.

FGD and IDI guides (included in a Supplement) included topics regarding care-seeking behaviors, perceptions of health services, and recent interactions with the health system. All participants gave their written informed consent before data collection. The local research team collected the data in French or 1 of 4 local languages (Malinké, Kpele, Soussou, or Poular) and then translated and transcribed the data in French.

A team of 4 researchers, all fluent in French, conducted a thematic content analysis using both inductive and deductive approaches to develop and refine the codebook. One of the lead researchers wrote the field report and identified preliminary themes from the FGDs and IDIs. Two other researchers read a set of transcripts and wrote memos to identify further concepts. Four team members then coded the same 2 transcripts, discussed discrepancies, and added additional codes for emergent concepts as needed. Using the revised codebook, the team then double-coded 2 transcripts of each type of interview or FGD (8 transcripts, or 12% of the total number of transcripts), in pairs to ensure consistency of the coding process. The pairs agreed, by consensus, upon the final coding for these 8 transcripts and made revisions and clarifications to the final codebook accordingly. Following this consensus process, team members then coded the remaining transcripts with the revised codebook. The full data analysis team applied a codebook—informed by these memos, field report, and original study objectives—to code French transcripts using Atlas.ti software.^22^ Following coding, the team further analyzed the coded data to generate crosscutting themes.

### Ethics Approval

The Johns Hopkins Bloomberg School of Public Health Institutional Review Board (IRB#6739) and the Guinean ethics committee (Comité National d’Ethique pour la Recherche en Santé, IRB#: 71/CNERS/16) approved the study. The CNERS is a group of experts convened by the Ministry of Health who review protocols for health-related research in Guinea.

## RESULTS

A total of 283 individuals participated in the study across 24 FGDs and 45 IDIs (Table 2). An average of 10 community members participated in each FGD.

**TABLE 2.** Study Participants, Qualitative Study on RMNCH Service Utilization, Guinea

**Subpopulation**	**Number of FGDs/IDIs**	**Number of participants**
Mothers of children aged younger than 5 years	29 IDIs	29
Health care providers	16 IDIs	16
Grandmothers of children aged younger than 5 years	12 FGDs	118
Male heads of household	12 FGDs	120
Total participants		283

Abbreviations: FGD, focus group discussion; IDI, in-depth interview; RMNCH, reproductive, maternal, neonatal, and child health.

In the following sections, we explore how trust influenced care-seeking decisions, which specific aspects of trust emerged as most relevant to community members, and what recommendations surfaced for improving trust in health workers and the health system.

### Trust and Care Seeking

Trust in health systems and specifically within the client-provider relationship emerged from FGDs and IDIs across rural and urban areas and among all types of respondents as a central element of care-seeking decisions. A clear link between trust and the willingness to seek care surfaced. A grandmother in Friguibegbe delivered her children in the hospital and encouraged her own children to give birth in the hospital because “*it is the hospital we trust*.” Likewise, providers were aware of the connection.

*It is when my behavior frustrates the sick, he will no longer have trust in me, and he will not return to me again. —*Health worker, Télimelé

In FGDs and IDIs, trust in health systems and with the client-provider relationship emerged as a central element of care-seeking decisions.

Comments from participants in the study confirmed that mistrust in providers was directly related to the Ebola crisis, particularly the fear of being diagnosed with Ebola and the persistent belief that providers were deliberately or through negligence spreading Ebola.

*Before Ebola, the* [frequentation] *of our health center was normal and there was absolute trust between us and our doctors. But during Ebola, the frequentation was not there anymore. Everyone was afraid to be considered an Ebola patient or to catch the disease. —* Mother, Nzérékoré

*Before Ebola, people normally frequented our health center and trust existed between patients and providers, but during Ebola people refused to seek treatment at the hospital because they were afraid of being killed … Today our health center became our own slaughterhouse. After Ebola to the present, trust between the community and the doctors does not exist. —*Male head of household, Bendou

Providers were also aware of trust issues.

*We had lost the trust of the population; the patients could tell you that they did not have trust in us during Ebola. On the contrary, they thought we were paid to kill them. —*Health worker, Télimelé

Though many community members, particularly male heads of household, expressed negative attitudes toward providers since Ebola, community members and providers alike expressed a desire for trust to return.

*The most important thing is the strengthening between the community and the health providers. It is crucial that there is trust between these 2 camps. —*Male head of household, Ratoma

Though many community members expressed negative attitudes toward providers since Ebola, community members and providers alike expressed a desire for trust to return.

Participants in the study also identified signs of improvement post-Ebola.

*With the end of Ebola, trust between health providers and patients is beginning to return little by little. —*Male head of household, Ratoma

*During the Ebola virus illness, I did not go to the health center because everyone said there was the source of contamination. But now I’m starting to go there because trust is returning.* —Mother, Télimélé

While these types of rumors circulated during the Ebola epidemic that health centers were deliberately or inadvertently infecting clients, participants like this mother described an improvement in the aftermath. Specifically, respondents considered changes in infection control and cleanliness at health facilities to be positive and lasting outcomes of the Ebola epidemic.

*The passage of Ebola was an opportunity in the sense that it brought a positive change, since the arrival of Ebola and to this day, there are handwashing kits and thermometers in all the health structures and schools. —*Male head of household, Ratoma

### The Client-Provider Relationship: Expectations and Constructs Linked to Trust

As participants reflected on the client-provider relationship during and after the epidemic, there was a clear expectation that providers have an intrinsic duty or obligation to treat clients well, exemplified by the oath they had taken as medical professionals. This perception of duty, arising from their role as health workers, was expressed by community members—particularly male heads of household—and health workers alike. Community members expressed a strong sense that providers had an obligation to welcome clients, take good care of them, and give effective medications.

*What is first and foremost the good work of a doctor is knowing that he is there for the patients. —*Male head of household, Télimélé

Study participants placed special importance on the oath taken by providers to save lives. From the perspective of the community members, this oath represented a core obligation, specifically for doctors, that was integral to their role as doctors.

*They are called doctors, they must save lives, it is why they take the oath. And they have to respect this oath. —*Male head of household, Senko

Respondents perceived provider behavior that contradicted this oath to be a betrayal of the basic foundational duty to heal and spoke of it like a broken promise.

*But if you do not have money for your care, do not go, at the risk that they will ridicule you. The health providers, in general, behave like they haven’t taken an oath to save lives. —*Male head of household, Ratoma

Providers shared a similar understanding of the obligation incurred by taking the oath.

*It’s a purely social service, a humanitarian service, we were sworn to provide this service.* —Laboratory worker, Senko

With the perceived duty to treat as the expected foundation to a trusting client-provider relationship, respondents further described the following characteristics as influencing trust in health workers: (1) competence, (2) hospitality, (3) compassion, (4) honesty, and (5) confidentiality.

Respondents stated that trust in health providers was influenced by providers’ competence, hospitality, compassion, honesty, and confidentiality.

#### 1. Perceived Competence of the Provider

For respondents, competence meant that the treatment provided during the consultation was effective.

*The last time I went to the health center, the medications they prescribed me to buy treated my illness, so I can say that the health workers there are competent*. —Mother, Friguibegbe

Along with effective medication, the ability to give a correct diagnosis was also an essential aspect of competence, according to community members. The perceived competence of providers played a central role in individuals’ care-seeking decisions.

*It’s based on the technical competence of the staff that I choose my place of treatment.* — Male head of household, Bendou

Providers also understood this link between competence and care seeking. They were aware that their own skills had the power to build trust and promote the use of services.

*Quality service is when the patient is welcomed well, they find the place clean, the health workers well trained and competent. If there is knowledge, competence, and trust, the patients will use the health facilities.* —Health worker, Télimélé

#### 2. Perceived Hospitality at the Facility

Both health workers and community members emphasized the importance of hospitality to establishing and ensuring trust. A participant described suffering from severe malaria:

*The thing that made me feel at ease discussing my concerns and my health problems is the trust I have in my doctor. This doctor is a trustworthy man who keeps medical secrets, who is welcoming.* —Grandmother, Bendou

A key component of a warm welcome was listening; clients wanted providers to ask them questions or to consult with them.

*I want, when arriving at the hospital when I’m sick, that the doctor will welcome me after I greet him. When he finishes welcoming me, I describe my health problem, the development of my illness*. —Male head of household, Nzérékoré

Participants expressed a desire to explain their health problem themselves, to tell their story in their own words.

Similar to clients, providers also emphasized hospitality as integral to building trust.

*It is the way the client is welcomed: he is treated confidentially, the smile the doctor has, the trust he places in the doctor.* —Health worker, Télimélé

Providers linked their behavior, particularly regarding a warm welcome, with promoting a good image for themselves and their facility. Failing to offer a warm welcome could prove damaging.

*Now that relates to our weaknesses, for example, because if we are not welcoming, we risk tarnishing the very image of the facility.* —Health worker, Ratoma

Despite this shared perception between community members and health workers about the importance of hospitality, some participants expressed that clients continued to feel unwelcome upon arrival at the facility. Male heads of household were particularly critical of the welcome, not only on their behalf but on behalf of family members.

*Recently, my grandmother was sick, but frankly, we were poorly received*. —Male head of household, Friguibegbe

Poor welcome often influenced the decision to return to a health facility and at times sent clients to private clinics or traditional healers instead of public facilities.

#### 3. Perceived Empathy of Providers Toward Clients

Empathy encompassed the expectation that providers would show concern, feel compassion, or attempt to reassure or console patients who were afraid. Study participants expected health workers to “have pity” and be “humane,” to “interest themselves” in clients, and to “share the suffering” of clients, to “approach” clients, or to “worry” about their clients. Community members considered compassion to be part of the job of providers and a way for providers to inspire trust.

*The doctor must know that the patient suffers when he comes to the hospital, so the doctor must share this feeling of suffering with the patient and approach him by giving him the necessary advice and care. That gives him moral strength and makes the patient trust in his healing.* —Grandmother, Nzérékoré

*If you intend to return a patient to perfect health, you must first approach him and express feelings of compassion. Here it does not happen like that.* —Male head of household, Bendou

The loss of trust because of providers shouting at or insulting them was keenly felt.

*If a doctor is someone who always shouts at patients, others will truly lose hope and trust in the doctors.* —Grandmother, Senko

Empathy often encompassed respect, welcome, and effectiveness of care, illustrating the idea that compassion may be both affective and effective. It is affective in that it is a nontechnical, emotional aspect of a relationship, even connected to love itself.

*Those who loved you and had pity on you, respected you.* —Male head of household, Télimélé

Compassion is effective—and linked to competence—in that it can produce relief from sickness. The belief that empathetic treatment would improve the patient’s physical health, even without medical treatment, revealed a holistic view of healing that extends beyond biomedical effectiveness.

*At the hospital, when the doctor welcomes the patient, encourages him, sympathizes with his pain and speaks to him with kind words, this constitutes for him the first treatment one can give him.* —Grandmother, Ratoma

*When the patient trusts me, he has the conviction that I want to satisfy him. When the patient knows that his anxiety can be lifted by me, the pains can be treated by me with a confidence, with a particular love, truly he will come back. But when I shout at the patient and do not listen to him and answer maliciously, the patient will not return anymore.* —Health worker, Télimelé

Providers shared the view that empathy was an important part of the treatment process and observed that clients who did not experience empathetic care at the clinic may not return.

#### 4. Perceived Honesty

Community members expressed fears that during the Ebola epidemic, providers falsely diagnosed patients with Ebola and/or deliberately infected patients with the virus. In fact, the main way that participants framed dishonesty as a barrier to trust was the belief that providers were secretly and intentionally infecting patients with Ebola. This perception of dishonesty was associated with a fracture in trust.

*The vaccination campaigns organized by the health personnel were an easy route to contaminate a lot of people and kill them if they wanted and earn money. They also said that the medicines that the doctors gave at the hospital contained the Ebola virus, and there was a complete and total rupture in trust between the community and the doctors.* —Male head of household, Bendou

Community members also described honesty regarding financial concerns as important to the client-provider relationship. They expressed a desire for transparency about the cost of services and products and cited examples of health workers stealing medications from patients or from the hospitals to use in their own private clinics or to sell. Men, in particular, differentiated between access issues—the true cost of providing care and whether they could afford it – as opposed to honesty issues—the perceived tendency of providers to extort clients for extra money.

*Usually, I go to health centers to get answers to my health concerns, but at present I am discouraged because the providers extort us at the health facilities.* —Male head of household, Ratoma

Community members also described honesty regarding financial concerns as important to the client-provider relationship.

As with many components of trust, participants emphasized that financial honesty influenced care-seeking decisions.

*Even before Ebola came to Guinea, the doctors’ behavior did a lot, so a lot of people no longer went to the hospital. Because they extort patients, there is no welcome, all* they *know is money. So many people no longer went to the hospital. —*Male head of household, Nzérékoré

Many participants likewise differentiated between expense and extortion in their willingness to seek care at health facilities.

#### 5. Confidentiality

For participants, confidentiality meant that providers would not disclose personal medical information—neither diagnosis nor treatment—with others.

*My health provider should keep my medical secrets, that is to say he should not tell anyone else about my diagnosis or the medicines he prescribed me. —*Mother, Nzérékoré

Some community members shared positive experiences with confidentiality and expressed the belief that providers were guarding their medical information. From the provider perspective, confidentiality was a way to relieve clients and further build trust.

*Trust between the patient and me, discretion between the patient and me. —*Health worker, Télimélé

### Keys to Rebuild Trust: Communication and Community Mobilization

Both health workers and community members repeatedly identified communication and community mobilization as key to rebuilding trust and ultimately restoring health facility utilization.

*Communication is a key factor to have trust between the provider and users*. —Health worker, Senko

A community member described community mobilization as a precursor to increased service use.

*It’s the community health worker and the doctors that we have here, that sensitized us and told us every time that the Ebola illness is gone, so come to the hospital. It’s because of this that people are beginning to return anew to the hospital. —*Male head of household, Senko

A community member described community mobilization as a precursor to increased service use.

However, one health worker discussed some of the challenges in community mobilization.

*It’s the sensitization that is very difficult, especially with the arrival of Ebola. Sometimes they insult us, but we always hold on to this because we work for our community, even if they don’t trust us anymore. —*Health worker, Télimélé

While recognizing the trust breakdown and even describing mistreatment, health workers emphasized the importance of continuing engagement and communication, noting the effect of these activities on rebuilding the relationship between individuals and the health system.

### Program Adaptation

HC3 incorporated current study findings into Gold Star campaign messages and program strategies in Guinea, particularly the development of interpersonal communication trainings for health providers. Study findings also informed the project in the development of community dialogues between community leaders and health workers to encourage better mutual understanding of the challenges faced by each group and to develop shared solutions to address those challenges. At the community level, people were invited to attend “open-door days” at their local health facilities, where they could visit with providers to learn about the services offered and the associated costs to build trust in the competence and financial honesty of the clinic.

Since HC3 ended, a new iteration, Breakthrough ACTION, began. In 2020, the project began efforts in COVID-19 response and then in 2021 around Ebola. The team is working to identify ways to apply insights around trust from this study into both programmatic areas.

## DISCUSSION

Findings from the current study confirmed that trust was critical to overcoming the client-provider gap in post-Ebola Guinea, especially as community members began considering whether to return to health services. Through IDIs and FGDs with mothers and grandmothers of young children as well as male heads of household and health workers, participants conceptualized client-provider trust in terms of their previous health care experience, how trust changed during and after the Ebola epidemic, and how trust in the health system could be restored. Study participants linked health workers to negative experiences during the epidemic and reported that they consequently avoided health facilities. These findings are similar to other studies that noted a reduction in the use of services throughout the epidemic.^6^^,^^23^

Study findings also illustrated—consistent with other literature^24^^–^^26^—that in the post-Ebola Guinea context, both technical (competence) and nontechnical (compassion, hospitality, honesty, confidentiality) aspects of care contributed to a trusting relationship. Participants also highlighted the constellation of factors that comprise quality, trustworthiness, and client satisfaction, which overlap with those shown to be important outside of West Africa such as perceived knowledge and expertise, perceived openness and honesty, and perceived concern and care.^17^ There were, however, divergences from domains identified as relevant in other settings, such as provider agency or perceived fairness.^18^^,^^27^ Notably absent in discussions of trust were access issues. Although participants did describe a variety of access issues—the cost and availability of medications, the presence of personnel at the clinics, and the distance to the facility—they were rarely linked to trust.

Findings showed that in the post-Ebola Guinea context, technical and nontechnical aspects of care contributed to a trusting relationship.

In the current study, the perception that providers have an obligation to treat patients well framed core expectations around the provider-patient relationship. Respondents strongly emphasized the duty to treat clients even at the cost of one’s own self-interest. The frustration and indignation expressed by participants regarding negative experiences at facilities were inconsistent with a cynical and resigned posture toward the health system. Both health workers and community members alike felt that taking the oath to save lives is meaningful, and anything short of care in line with that oath was a betrayal. These beliefs about the duty of the provider reveal that, despite years of weakened health systems from civil wars in the Mano River region and from the more recent Ebola epidemic, an underlying idealism persists about the way the health system and service provision should function.

In general, the extent of overlap between clients and providers in describing the key elements of a trusting relationship was notable. It was not the case that providers were unaware of the existence or causes of the breakdown in trust. Although the emphasis was slightly different— with providers tending to emphasize hospitality, compassion, and competence while deemphasizing honesty—the constructs themselves were largely shared. In general, both providers and clients identified communication as a key pathway for overcoming the boundary between them. Comments from both community members and providers suggested a desire for more dialogue and 2-way communication. Increased attendance at health facilities within the project’s catchment areas suggests that the insights gained from the current study made for wise programmatic decision making.^28^ Although attributing the increase in health facility attendance to the project’s efforts is beyond the scope of the current study, these trends, nevertheless, suggest the potential value in investing in and applying quality formative research and call for further rigorous evaluation of the impact of such programs in the future.

The importance of trust in epidemic control and recovery identified in this study is consistent with other studies.^10^^,^^13^^,^^29^^,^^30^ For example, Blair et al. conducted a representative survey in Monrovia, Liberia, to gauge trust in the government and international NGOs to control the epidemic, linking reported trust to the willingness of participants to comply with epidemic control measures.^11^ Similar quantitative studies are needed as countries progress in the COVID-19 response and as Guinea faces Ebola once again. A multicountry set of surveys conducted via social media suggested that in the WHO Africa region, fewer than 60 percent of survey respondents trusted health workers as a source of COVID-19 information.^31^ Future surveys could investigate nuances of trust-related constructs to inform decision making for COVID-19, Ebola, and similar public health emergencies.

Overall, study findings illustrated that trust was a salient construct linked to health care service utilization in post-Ebola Guinea. Moreover, improving client-provider trust in a postemergency setting may accelerate a return to health services. These insights, although obtained in the context of 1 specific public health project, can serve other programs dealing with health emergencies, such as what we currently see with COVID-19. Moreover, the recent resurgence of Ebola in Guinea has raised concerns throughout West Africa and reemphasizes the value of this study. The study findings underscore the importance of identifying the key leverage points for trust in health systems and providers to rebuild trust and restore health care service utilization.

The study findings underscore the importance of identifying the key leverage points for trust in health systems and providers to rebuild trust and restore health care service utilization.

### Limitations

It is important to highlight the limitations of this study. First, researchers did not ask participants to explicitly define trust or the specific constructs, so the research team was unable to analyze how participants directly described or defined trust. The base of the current analysis stemmed from mentions of trust in the context of sharing experiences with the health system and perceptions of quality services. At the same time, because the study was not explicitly asking about trust but rather exploring perceptions of quality of care and health care service utilization, the unsolicited importance and salience of trust among community members and health workers is compelling. The lack of direct questioning about trust allowed for a more inductive approach to analysis, identifying the importance of trust and its corresponding components built from participants’ spontaneous expressions. Second, while all the study sites were affected by Ebola, they were not all affected to the same extent. In terms of the total number of confirmed Ebola cases, Conakry was most affected, followed by Nzérékoré and then the other sites.^32^ Therefore, this study cannot speak to perceptions of trust under “normal,” or nonepidemic, circumstances. As there was little variation across geographic areas, it is plausible that the constructs associated with trust and the client-provider relationship may be applicable in nonepidemic-affected settings as well. Nevertheless, the study’s primary focus was to explore a postemergency context and the findings can benefit programs aiming to encourage health care service utilization in a similar setting or predicament.

## Supplementary Material

21-00429-Tibbels-Supplement.pdf

## Translation

**En Français**

**L'importance de la Confiance dans la Relation Client-Prestataire en Guinée après Ebola: Resultats d'une Etude Qualitative**

**Principaux Résultats**

- La confiance est un déterminant essentiel de la recherche de soins de santé et devrait être une considération clé pour les programmes de santé mondiale opérant dans des contextes de post-urgence.
- La confiance est un composite de divers domaines sous-jacents, et le rétablissement de la confiance dépend de la compréhension et de la prise en compte des domaines qui sont importants pour les clients.
- Cette analyse présente une étude de cas sur la façon dont un programme de santé mondiale a utilisé la recherche qualitative pour explorer les principaux domaines de confiance dans la relation client-prestataire en Guinée en période post-Ebola et a développé des interventions pour aborder ces domaines.

**Principales implications**

- Pour améliorer les pratiques de recherche de soins lorsqu'il existe des barrières de confiance entre les communautés et le système de santé, les responsables de programmes doivent chercher à mettre un accent particulier sur les clients en définissant et en abordant leur conception de la confiance.

**Résumé**

Le manque de confiance dans le système de santé peut constituer un obstacle à l'utilisation des services, en particulier dans les contextes de pandémie et de post-urgence. Bien que des recherches antérieures aient identifié les domaines de confiance qui contribuent à la confiance des individus dans le système de santé, peu de recherches existent dans les pays à faible revenu ou intermédiaire, en particulier pendant et après les épidémies de maladies infectieuses. La présente étude, menée pour donner des informations les activités d'un programme post-Ebola, a exploré les perceptions et les expériences de la prestation de soins de santé en Guinée dans la période post-Ebola, en accordant une attention particulière à la confiance. Les chercheurs ont mené des entretiens approfondis avec des agents de santé (n=15) et des mères de jeunes enfants (n=29), ainsi que 12 discussions de groupe avec des grands-mères de jeunes enfants et 12 avec des hommes chefs de famille. L'étude s'est déroulée en Basse Guinée et en Guinée Forestière, deux (2) régions les plus durement touchées par Ebola. Les personnes interrogées ont identifié un abus de confiance pendant l'épidémie, et plusieurs domaines sont apparus comme pertinents pour le renouvellement de la confiance et des pratiques de recherche de soins. Au cœur d'une relation de confiance client-prestataire se trouvait la conviction que les prestataires avaient le devoir intrinsèque de bien traiter les clients. Ainsi, la compétence perçue du prestataire, l'hospitalité de l'établissement, l'empathie du prestataire, la transparence des coûts et l'engagement envers la confidentialité sont apparus comme des influences pertinentes sur la confiance des participants envers les prestataires. Les membres de la communauté et les prestataires ont exprimé des points de vue similaires concernant la confiance et ont discuté du rôle de la communication ouverte et de la mobilisation de la communauté pour rétablir la confiance. Les conclusions de l'étude ont éclairé diverses activités du programme, notamment l'élaboration de messages de campagne et les formations à la communication interpersonnelle pour les agents de santé. Cette étude fournit des informations précieuses sur certaines composantes sous-jacentes de la confiance qui peuvent constituer des points de levier clés pour rétablir la confiance et promouvoir la recherche de soins dans les situations de post-urgence. Ces informations éclairent les activités du programme dans la réponse actuelle à Ebola en Guinée et pourraient être utiles dans d'autres crises, comme la pandémie mondiale de coronavirus (COVID-19).

**Peer Reviewed**
