# Supplementary material for: The Salience of Trust to the Client-Provider Relationship in Post-Ebola Guinea: Findings From a Qualitative Study
Source: Glob Health Sci Pract. 2022 Feb 28;10(1):e2100429. doi: 10.9745/GHSP-D-21-00429 (PMC8885337; doi:10.9745/GHSP-D-21-00429)
Supplement: 21-00429-Tibbels-Supplement.pdf [file 21-00429-Tibbels-Supplement.pdf]

Supplement to: Tibbels N, Hendrickson Z, Mills H, Sidibé S, Vondrasek C, Gurman T. The salience of trust to the client-provider relationship in post-Ebola Guinea: findings from a qualitative study. Glob Health Sci Pract. 2022;10(1):e2100429. https://doi.org/10.9745/GHSP-D-21-00429

In-depth interview guide: Mothers (ages 20-40) of Children Under Age 5

Note: Italics are guidelines to the interviewer and are not to be read aloud.

1. First I would like to get to know you a little. To begin, I would like to hear about a typical day for you. What do you do from sunrise to sunset? *(Explore things such as the following:*
   - *who lives with them*
   - *type of activities they do during the day*
   - *where they go during the day*
   - *how much time they spend at various activities*
   - *how many kids they have*
   - *when they go to market*

2. When you have a question or concern about health, where do you go to get answers? *(Explore various types of communication channels. You don't necessarily need to read through the list below. But we want to make sure that if the channel has not been mentioned, to ask about it specifically)*
   - Radio?
   - Television?
   - Print (ie newspaper, billboards)?
   - Internet/social media?
   - Specific people? (i.e. health care providers, husband, mother-in-law, friends, other family members)

3. When your child is sick, how do you decide where and when to seek help?
   - Walk us through how you manage your care when your child is sick?
   - Receiving treatment including finding and asking permission, decision-making related to treatment-seeking (*preventive versus curative*)
   - How does this change for prevention services such as immunizations?
   - What is your first line of health care for your child? What do you do? Where do you go first? With whom do you talk to first?

4. Please tell me about the last time you or your child was sick. What was the situation?
   - What was the first thing that you did? And after that?
   - How did this situation affect your daily routine that you told me about before?

5. No I am going to ask you some questions about the last time you went to the local health center? *(Get a sense of the illness/condition and how they handled it)*

- How would you describe the doctor/nurse/other staff at the health center?
- How was the welcome?
- What were things that you liked about your visit?
- How were you made to feel?
- Did you feel like your wellbeing mattered to them?
- What did they do to make you feel this way?
- What were some of the good things you experienced? Bad things?
- What could have been done to make your visit better?
- What could the local health center do to make a woman feel more at ease?

6. Would you recommend your local health center to your friends? Why or why not?

7. When you were last pregnant, how did you decide where and when to seek help?
   - Walk us through how you managed your pregnancy?
   - Receiving treatment including finding and asking permission to go to ANC services, decision-making related to ANC service attendance?

8. What does it take for you to decide to seek care either at the hospital or the local health center?
   - When/for what do you go to a local health center?
   - When/for what do you go to the hospital?
   - When/for what do you go to the community health worker?
   - When do you think other people choose the hospital versus the health center versus a community health worker?

9. How do you choose where you go for health care services?
   Possible probes:
   - *appearance/cleanliness*
   - welcoming environment of the health center
   - friendly staff
   - technical competence of the service providers
   - health care provider that is kind/treats patients with respect
   - *cost*
   - *hours*
   - *location*
   - *ability to communicate in my language*
   - *protection from infection*
   - *recommendation from friend/family member*
   - *etc.*

In-depth interview guide: Mothers (ages 20-40) of Children Under Age 5

10. What do you think of the health services offered at your local health center?
    Possible probes:
    - *appearance/cleanliness*
    - welcoming environment of the health center
    - friendly staff
    - technical competence of the service providers
    - health care provider that is kind/treats patients with respect
    - *cost*
    - *hours*
    - *location*
    - *ability to communicate in my language*
    - *protection from infection*
    - *recommendation from friend/family member*
    - *etc.*

11. What do you expect to get in quality services?
    - How would you define quality services ? What does that term mean to you ?
    - What do you want to have in quality services ?
    - What do you think other people think of the local health center?
    - What do you think other people expect in terms of quality of services?

12. I am going to ask you a few questions about health care providers and staff at the health center.
    - How would you like providers to treat you?
    - How would you like staff to treat you?
    - Some people say that they like to be treated with respect when they go receive health care services. What does respect mean to you ?
    - What should HC providers do to fight infection?

13. If you were to compare your health center to a type of cloth/piece of clothing, what type would it be and why ?
    - What do you think other people in your town would say ?
    - You described the local health center as ____________ (*summarize the characteristics/traits the respondent used to describe the health center in the question about the piece of clothing as well as other questions regarding their experience with the health center).* How have your views of the health center changed, if at all, since Ebola?
    - You said that people in your town perceive the local health center as ____________ (*summarize how the respondent described the health center in Question 11 above about the health center).* How do you think their views of the health center have changed, if at all, since Ebola?

In-depth interview guide: Mothers (ages 20-40) of Children Under Age 5

14. Is there anything else that you would like to add?
    (Give the respondent an opportunity to add any final thoughts.)

Thank you for your time!

In-depth interview guide: Health care providers

1. First I would like to get to know you a little. *(Please note gender in your notes.)*
    - Age
    - Language/s spoken
    - Ethnic group
    - Profession
    - Work setting
    - Number of years in profession

2. Please tell me about your job/profession as well as your role here at the hospital/health center/community.

    Possible probes:
    - *Role at hospital/health center/community*
    - *What type of services he/she provides*
    - *Type/range of clients interact with most often (ie all community members, women)*
    - *How long have been employed at this particular facility*
    - *Get a sense of other interactions, responsibilities, etc.*

3. Please tell me about a typical health care consult that you might have with a client/patient.
    - What is the process from when you first go to meet a patient to when the visit is finished?
    - When a client leaves, how do you know if he/she is pleased with the care received?

4. What would you say is the most rewarding thing about being a ______________ *(specify type of provider that respondent is)*?
    - What would you say is the most rewarding thing about being a _________ *(specify type of provider that respondent is)* at this hospital/health center/community?

5. What would you say is the hardest thing about being a ____________ *(specify type of provider that respondent is)*? ?

    What would you say is the hardest thing about being a ____________ *(specify type of provider that respondent is)* at this hospital/health center/community?

6. How well do you think the local health center serve the needs of the people who might seek care there?
    - Women?
    - Children?
    - Community in general?
    - How has the quality of services changed since the ebola crisis?

In-depth interview guide: Health care providers

7. As I mentioned before, we are working with the Ministry of Health. Part of the work involves working to establish quality services. When you think about quality services, what does that look like for you? What does quality mean to you?

8. I am going to ask you a few questions about things that might affect the quality of care you are able to provide patients.
   - What skills, materials, information, or support do you feel you need in order to better serve the community?
   - *(Ask only for providers who work at local health center)*How does the local health center, in policy and in practice, **support** quality of services?
   - *(Ask only for providers who work at local health center)*How does the local health center, in policy and in practice, **hinder** quality of services?
   - *(Ask only for providers who work at local health center)*In what ways does the local health center support your ability to provide quality services? *(e.g. support staff, health education materials, job aids, professional development trainings)*
   - What do you feel that you need/want from patients/community members?
   - What could patients/community members do to make it easier for you to serve them?

9. What are your biggest strengths you have when it comes to serving the community/providing health care services?
   - What are your biggest weaknesses you have when it comes to serving the community/providing health care services??
   - What is the single most important thing the health center could do to better meet the needs of the community?
   - What is the single most important thing that you as a health care provider could do to better meet the needs of the community?

10. Given all that you have shared with me today, I want to end with a question that asks you to be a little creative. If the health center was a car, what kind of car would it be, and why?
   - How have your views changed, if at all, since Ebola?
   - If I asked patients who come to the health center the same question, how do you think they would answer?
   - How do you think their views of the health center have changed, if at all, since Ebola?

Is there anything else that you would like to add?
(Give the respondent an opportunity to add any final thoughts.)

Thank you for your time!

Focus group discussion guide: Male heads of households (ages 20-50) with Children Under Age 5

Opening 10 minutes

A. Introduction
- Thank you for coming!
- Introduction of facilitator/notetaker and CCP
- Your presence and participation matter to us

B. Purpose
- Explain why they are here- describe focus group
- The purpose of this group discussion is to learn about the places people like you go for health care, your experiences in regards to the care you have received, and your thoughts about your local health center.
- Your feedback and input is very helpful to us. We will be using it to help us in our work with local health centers to improve quality of care they provide.

C. Expectations

Before we get started, I want to explain a few things about how we will work during our time together:
- As you have been told before, you can choose whether or not you wish to talk . You can also choose to leave at any point during our time talking.
- We will be on first name basis tonight (hence the name tags), but names will not be included in our final report
- I will be recording our discussion to make sure that I do not miss any comments. I will be the only person that will listen to the recording, which I will use when I type up what we discussed.
- Please speak one at a time and speak up (especially since recorder will garble voices if too many speak at once) but you do not have to wait for me to call on you.
- Feel free to express when you do not agree with something someone said, as we are trying to gather many viewpoints—there are no right or wrong answers. Please do not forget to respect what other people say. Also, make sure others get a chance to speak.
- We have a lot to cover in a short period of time, so I will be moving from one thing to the next somewhat quickly. If you have something you want to add and I move on, please feel free to stop me and express your thoughts.
- We will talk for no more than 2 hours.

Warm-up 10 minutes

1. Please share with us the following:
    - First name
    - Age
    - Where you live and with whom
      Your favorite thing about living in your town/village

Focus group discussion guide: Male heads of households (ages 20-50) with Children Under Age 5

General questions 15 minutes

2. When trying to get answers about health concerns/questions where do you go? *(Explore various types of communication channels. You don't necessarily need to read through the list. But we want to make sure that if the channel has not been mentioned, to ask about it specifically)*
    - Radio?
    - Television?
    - Print (ie newspaper, billboards)?
    - Internet/social media?
    - Specific people? (ie health care providers, friends, other family members)

In-depth questions 40 minutes

3. Now that we have a better idea about effective ways to get information to you as well as other, we are going to narrow in a little more and look at places where you go for health care.
4. Please think of 3 words or phrases that describe how you want your family to be treated when they go for a health care visit. [GIVE A MINUTE] Let's share.
5. Think back to the most recent visit your family made to the health center. What did you hear about the experience?
    - What went well? Explain.
    - What could have gone better? Explain.
6. When you or a family member has needed to go to a clinic/health care facility, when do you decide that it is time to go?
7. Once you have decided to seek care for either yourself or a family member at a clinic/health care facility, how do you choose where to go?
    Possible probes:
    - *appearance/cleanliness*
    - welcoming environment of the health center
    - friendly staff
    - technical competence of the service providers
    - health care provider that is kind/treats patients with respect
    - *cost*
    - *hours*
    - *location*
    - *ability to communicate in my language*
    - *protection from infection*
    - *recommendation from friend/family member*
    - *etc.*

8. What are the reasons you or members of your family would choose to go to the local health center or maternity in particular? *[Keep this question open ended and let participants provide their answers.]*
    Possible probes:

Focus group discussion guide: Male heads of households (ages 20-50) with Children Under Age 5

- *appearance/cleanliness*
- welcoming environment of the health center
- friendly staff
- technical competence of the service providers
- health care provider that is kind/treats patients with respect
- *cost*
- *hours*
- *location*
- *ability to communicate in my language*
- *protection from infection*
- *recommendation from friend/family member*
- *etc.*

9. What influences your decision as to whether or not you decide to return the next time you need health care services?

10. What do you think of the health services at your local health center?
    - How have perceptions changed, if at all, since Ebola?

11. When you think of quality services, what comes to mind?
    - What does quality mean to you?
    - What do you expect to get in terms of quality of services?

12. Some people feel that in order to have high quality health services you need to have high quality health care providers. So now I want to spend some time thinking about what an ideal health care provider looks like.
    - Please go into groups of 2-3 people. *(Give time time for groups to form. Hand out markers and newsprint to pairs/triads.)*
    - Now I want your group to come up with what makes for a high quality health care provider and I want you to draw a health care provider with those qualities. *(Give groups 10-15 minutes to talk and draw their ideal health care provider.)*
    - Now I would like groups to briefly present their ideal health center to the larger group. *(Give each group 3-5 minutes to present their ideal health care provider. Make sure to collect each drawing and number according to the order it was presented to the group)*

13. Now I want to talk a bit about how you think that other people's opinions regarding health services. What do you think other people think of the health care services at the local health center?
    - How do you think other people's perceptions have changed, if at all, since Ebola?
    - What do people expect in terms of quality of services?

Focus group discussion guide: Male heads of households (ages 20-50) with Children Under Age 5

- When do people choose hospital versus local health center versus community health worker, etc?

14. In what ways is the local health center a welcoming place for the community?
    - What could the local health center do to make it a more welcoming place?
    - Describe how you know if providers can provide you or your family member with the care you/they need?
    - What should/do HC providers do to fight infection?
    - How respectful are providers? What does respect mean to you?
    - How would you like providers to treat you?
15. What could health care providers do to better serve your needs?As we start to wrap up everything we have talked about so far, I want you to take a moment and think about the local health center as a whole— its characteristics, qualities, strengths, weaknesses. Now, imagine that the health center is a car. What type of car would it be and why?
    - What are the specific characteristics of that car that are like the health center?
    - What do you think other community members would say?
    - Ideally, what type of car would you like the health center to be and why?

Closure 15 minutes

A. Summarize and synthesize findings and variety of opinions expressed in group—this allows for making sure what we heard is correct and allows individuals to clarify
B. Anything else that you want to say that has been unsaid?
C. THANK YOU for participating!!!

Focus group discussion guide: Grandmothers (ages 45-60) of Children Under Age 5

Opening 10 minutes

B. Introduction
- Thank you for coming!
- Introduction of facilitator/notetaker and CCP
- Your presence and participation matter to us

C. Purpose
- Explain why they are here- describe focus group
- The purpose of this group discussion is to learn about the places people like you go for health care, your experiences in regards to the care you have received, and your thoughts about your local health center.
- Your feedback and input is very helpful to us. We will be using it to help us in our work with local health centers to improve quality of care they provide.

D. Expectations

Before we get started, I want to explain a few things about how we will be working together throughout our discussions:
- As you have been told before, you can choose whether or not you wish to talk . You can also choose to leave at any point during our time talking.
- We will be on first name basis tonight (hence the name tags), but names will not be included in our final report
- I will be recording our discussion to make sure that I do not miss any comments. I will be the only person that will listen to the recording, which I will use when I type up what we discussed.
- Please speak one at a time and speak up (especially since recorder will garble voices if too many speak at once) but you do not have to wait for me to call on you.
- Feel free to express when you do not agree with something someone said, as we are trying to gather many viewpoints—there are no right or wrong answers. Please do not forget to respect what other people say. Also, make sure others get a chance to speak.
- We have a lot to cover in a short period of time, so I will be moving from one thing to the next somewhat quickly. If you have something you want to add and I move on, please feel free to stop me and express your thoughts.
- We will talk for no more than 2 hours.

Warm-up 10 minutes

1. Please share with us the following:
    - First name
    - Age
    - Where you live and with whom

Your favorite thing about being a grandmother of young children

Focus group discussion guide: Grandmothers (ages 45-60) of Children Under Age 5

- Describe where your grandchildren were born. (*Where and whether in the house or health care facility?*)

General questions 15 minutes

2. When trying to get answers about health concerns/questions where do you go? *(Explore various types of communication channels. You don't necessarily need to read through the list. But we want to make sure that if the channel has not been mentioned, to ask about it specifically)*
   - Radio?
   - Television?
   - Print (ie newspaper, billboards)?
   - Internet/social media?
   - Specific people? (ie health care providers, husband, friends, other family members)

In-depth questions 40 minutes

Now that we have a better idea about effective ways to get information to you as well as other, we are going to narrow in a little more and look at places where you go for health care.

4. I'd like for you to think of three words or phrases that describe how you want to be treated when you go for a health care visit. [GIVE A MINUTE] Let's share.
5. Think back to a health care visit where you felt at ease discussing your health concerns and problems. What about the experience made you feel comfortable?
6. Now think back to a health care visit where you **did not** feel at ease discussing your health concerns and problems. What about the experience made you feel **uncomfortable**?
7. Now I want to talk a bit about health care for your grandchildren. How are decisions made about when and where to seek health care services for your grandchildren?
8. What are the reasons you or members of your family would choose to go to the local health center? *[Keep this question open ended and let participants provide their answers.]*
   Possible probes:
   - *appearance/cleanliness*
   - welcoming environment of the health center
   - friendly staff
   - technical competence of the service providers
   - health care provider that is kind/treats patients with respect
   - *cost*
   - *hours*
   - *location*
   - *ability to communicate in my language*
   - *protection from infection*

Focus group discussion guide: Grandmothers (ages 45-60) of Children Under Age 5

- *recommendation from friend/family member*
- *etc.*

9. Some people feel that in order to have high quality health services you need to have high quality health care providers. So now I want to spend some time thinking about what an ideal health care provider looks like.
    - Please go into groups of 2-3 people. *(Give time time for groups to form. Hand out markers and newsprint to pairs/triads.)*
    - Now I want your group to come up with what makes for a high quality health care provider and I want you to draw a health care provider with those qualities. *(Give groups 10-15 minutes to talk and draw their ideal health care provider.)*
    - Now I would like groups to briefly present their ideal health center to the larger group. *(Give each group 3-5 minutes to present their ideal health care provider. Make sure to collect each drawing and number according to the order it was presented to the group.)*

10. Now I want to talk a bit about how you think that other people's opinions regarding health services. What do you think other people think of the health care services at the local health center?
    - How do you think other people's perceptions have changed, if at all, since Ebola?
    - When you think of quality services, what comes to mind? What does quality mean to you?
    - What do you expect to get in terms of quality of services?

11. In what ways is the local health center a welcoming place for the community?
    - What could the local health center do to make it a more welcoming place?
    - Describe how you know if providers can provide you or your family member with the care you/they need?
    - What should/do HC providers do to fight infection?
    - How respectful are providers? What does respect mean to you?
    - How would you like providers to treat you?
    - What could health care providers do to better serve your needs?

12. As we start to wrap up everything we have talked about so far, I want you to take a moment and think about the local health center as a whole— its characteristics, qualities, strengths, weaknesses. Now, imagine that the health center is a type of cloth. What type of cloth would it be and why?
    - What are the specific characteristics of that cloth that are like the health center?
    - What do you think other community members would say?

Focus group discussion guide: Grandmothers (ages 45-60) of Children Under Age 5

- Ideally, what type of cloth would you like the health center to be and why?

Closure 15 minutes

- Summarize and synthesize findings and variety of opinions expressed in group—this allows for making sure what we heard is correct and allows individuals to clarify
- Anything else that you want to say that has been unsaid?
- THANK YOU for participating!!!
